# Viral Metagenomics: Analysis of Begomoviruses by Illumina High-Throughput Sequencing

**DOI:** 10.3390/v6031219

**Published:** 2014-03-12

**Authors:** Ali Idris, Mohammed Al-Saleh, Marek J. Piatek, Ibrahim Al-Shahwan, Shahjahan Ali, Judith K. Brown

**Affiliations:** 1 Center for Desert Agriculture, King Abdullah University of Science and Technology, Thuwal 23955-6900, Saudi Arabia; E-Mails: marek.piatek@kaust.edu.sa (M.J.P.); shahjahan.ali@kaust.edu.sa (S.A.); 2 Department of Plant Protection, King Saud University, Riyadh 11451, Saudi Arabia; E-Mails: malsaleh@yahoo.com (M.A.S.); ialshahwan@yahoo.com (I.A.-S.); 3 School of Plant Sciences, The University of Arizona, Tucson, AZ 85721, USA; E-Mail: jkbrown@email.arizona.edu

**Keywords:** Illumina sequencing, geminivirus, ssDNA viruses, genome enrichment, viral genome assembly

## Abstract

Traditional DNA sequencing methods are inefficient, lack the ability to discern the least abundant viral sequences, and ineffective for determining the extent of variability in viral populations. Here, populations of single-stranded DNA plant begomoviral genomes and their associated beta- and alpha-satellite molecules (virus-satellite complexes) (genus, *Begomovirus*; family, *Geminiviridae*) were enriched from total nucleic acids isolated from symptomatic, field-infected plants, using rolling circle amplification (RCA). Enriched virus-satellite complexes were subjected to Illumina-Next Generation Sequencing (NGS). CASAVA and SeqMan NGen programs were implemented, respectively, for quality control and for *de novo* and reference-guided contig assembly of viral-satellite sequences. The authenticity of the begomoviral sequences, and the reproducibility of the Illumina-NGS approach for begomoviral deep sequencing projects, were validated by comparing NGS results with those obtained using traditional molecular cloning and Sanger sequencing of viral components and satellite DNAs, also enriched by RCA or amplified by polymerase chain reaction. As the use of NGS approaches, together with advances in software development, make possible deep sequence coverage at a lower cost; the approach described herein will streamline the exploration of begomovirus diversity and population structure from naturally infected plants, irrespective of viral abundance. This is the first report of the implementation of Illumina-NGS to explore the diversity and identify begomoviral-satellite SNPs directly from plants naturally-infected with begomoviruses under field conditions.

## 1. Introduction

Begomoviruses are small, circular, single-stranded (ss) DNA viral pathogens (family, *Geminiviridae*; genus, *Begomovirus*) of plants that infect a wide range of eudicots in the tropics and subtropics. The genome size of this genus ranges in size from 2.8 to 5.2 kb, and is arranged either in two components (bipartite) referred to as DNA-A and DNA-B, each approximately 2.6 kb, or in a single component (monopartite), approximately ~2.8 kb in size [1]. A number of begomoviruses endemic to the Eastern Hemisphere have a monopartite genome, and are associated with circular, ssDNA alpha- and beta-satellites that are about half the size of the genome of the “helper” begomovirus, at 1.4 kb, upon which they rely for aspects of the infection cycle [2,3]. Also, smaller than expected circular, ssDNA satellite molecules of ~0.7 kb in size are frequently detected together with begomoviruses in plant DNA extracts [4], as well as smaller than unit size (and variable in length) helper genome or satellite, some of which have been shown to function as defective interfering (DI) sequences [5]. However, the biological functions of DI DNAs with respect to the begomoviral infection cycle, virulence, or evolution are not well understood.

In most instances in which a helper begomovirus has been found to be associated with a beta-type satellite, it has been shown that a satellite is required by the helper virus to systemically infect the plant host, and to contribute to the development of characteristic disease symptoms [6,7]. These phenomena are in part made possible by the suppressor activity of the betasatellite-encoded protein, β*C*1 that silences the plant host response to viral infection [8]. Further betasatellites are encapsidated into virions for transmission from plant to plant, along with the helper virus, by the whitefly vector *Bemisia tabaci* (Gennadius) sibling species group [9,10]. 

The alpha-type of satellite on the other hand has been shown to be dispensable for systemic infection of the plant when the betasatellite and the helper virus are both present. Most experimental studies have used the plant viral-permissive host, *Nicotiana benthamiana* (Domin), instead of naturally infected plant species, resulting in the obfuscation of the possible role of associated satellites in the infection cycle [11].

Begomoviruses that are associated with DNA satellites represent a pathogen complex. Neither the ssDNA helper virus nor the betasatellite molecule encode a DNA polymerase, and so both must replicate in the plant nucleus using the host polymerase and a double-stranded DNA form by rolling circle replication (RCA) [12,13,14]. Recombination and mutations that occur during the replication cycle are important genetic processes that give rise to genomic and genetic variability presumably due to the ability to adapt to new environments, including host genetic variability [15,16,17]. The contemporary experimental approaches used for detecting and quantifying the extent of genetic variability within begomoviral genomes and the associated satellites employ either polymerase-mediated amplification (as much as 10,000 fold in a few hours) of viral genomic DNA by polymerase chain reaction (PCR) [18] using sequence-specific (or degenerate) primers, or phi29 DNA polymerase-mediated rolling circle amplification (RCA) through the use of random primers [19], followed by cloning and capillary DNA sequencing. However, these strategies produce limited information about the presumed predominant genomes, given that the depth of sequencing is low, that precludes assessment of the genetic differentiation and population structure of begomovirus-satellite variants. Indeed, the RCA or PCR amplification methods can be biased to unknown extents during early amplification steps that select for the most abundant viral and satellite DNA variants, and other potential artifacts of the methodologies.

Typically, the complete genome of monopartite or bipartite DNA components of begomoviruses and their associated circular, ssDNA satellites are cloned from the products of RCA [20,21] (commercial application available through GE Healthcare, Life Sciences, Piscataway, NJ, USA) or virus-specific PCR [21]. Also, begomoviral genome characterization has been achieved using a combination of RFLP (restriction fragment length polymorphism) and pyro-sequencing, referred to as “circomics” [22]. The amplification of begomoviral genomic and associated components by PCR, followed by cloning and capillary DNA sequencing, are limited by the specificity of the primers, and by the number of variants produced by the earliest amplification steps, and then by selection during the molecular cloning step. Typically, RCA produces high molecular weight products as dsDNA concatemers that are digested into unit-length components and cloned, with the inserts verified by capillary sequencing [19,23]. This is a time-consuming process, and in addition, a limited number of variants are represented among the resultant clones, based on the expectation that one or a few predominant genotypes are represented in the starting material. However, the innovative approach described here employs the robustness of the bacteriophage phi29 DNA polymerase used in RCA technology, together with deep sequencing using Illumina [24,25] and bioinformatics to assess population diversity of begomoviruses and their satellites in naturally infected plants.

For virus discovery from field samples at the population level, traditional methods such as RCA, or PCR followed by cloning and traditional DNA sequencing, are ineffective, particularly for detecting rare members in a population, or those containing minimal, single nucleotide (nt) polymorphisms in begomoviral populations, where mixtures of isolates, strains, and multiple species prevail. In addition, technical limitations can result in the inability to detect low- abundant begomoviral and/or associated DNA satellite molecules. In this study, circular, single-stranded DNA-containing begomoviruses and their satellites were enriched by RCA from total DNA extracts of naturally infected, symptomatic field plants. The enriched begomoviral genomes and satellites were subjected to Illumina-NGS, assembled, and subjected to analysis to detect polymorphisms.

## 2. Results and Discussion

The *de novo* assembly of the high-throughput Illumina 10 million (10M) reads was carried out using SeqMan NGen3 (DNASTAR Inc., Madison, WI, USA) software, and resulted in a large number of contigs that ranged from 247 and 2244 (Table 1). The contigs were used to search the NCBI-GenBank database to identify the most closely related begomovirus exemplars. The search result showed that the contigs containing begomoviruses and begomovirus-associated DNA satellite sequences consistently comprised the highest number of assembled reads, indicating that the RCA successfully enriched for these components.

**Figure 1:**
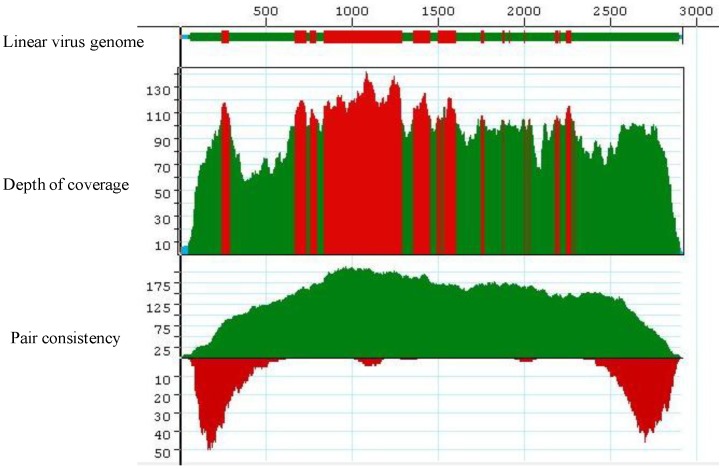
Complete coverage of the helper virus *Cotton leaf curl Gezira virus* (field sample KSA27) in reference-guided assembly. 100,000 Illumina short reads were used in this assembly. The red, green and cyan colors in the Coverage Threshold Graph for the linearized (with *ori* set as coordinate1) virus genome represent various levels of mapping of sequencing reads onto the reference viral genome, *Cotton leaf curl Gezira virus*. Red color exemplifies regions that exceed the maximum expected coverage level (set for 100 sequences at each position). The color green denotes regions sequenced on both strands that fell above coverage threshold (set for 4 sequences at each position). The color cyan denotes regions sequenced from only one strand. The colors in the Depth of Coverage graph represent the same regions as those in the Coverage Threshold graph. The pair consistency histogram was relatively low at the beginning and the end of the genome, an observation that could reflect the circular nature of the genome.

The preliminary *de novo* assembly of the resultant high-throughput sequence data (using the default setting) that consisted of 10 million reads, proved to be time-consuming for these relatively short genomic components, and further yielded a higher than necessary depth of coverage, and so was found to be unnecessarily wasteful (Table 1). This observation was confirmed by reducing the number of reads used in subsequent assemblies of both *de novo* and reference-guided assemblies to 100,000 (Table 1 and Table 2), from which results were similar to those using the 10M reads, while still achieving a adequate coverage (Figure 1).

**Table 1:** The *de novo* assembly statistics of enriched begomovirus genomes and satellite molecules.

Sample	Number of Contigs		N50 (kb)		Average Read Length (bp)		Average Phred Read Quality		Number of Assembled Viral Components
	10 M	100 K	10 M	100 K	10 M	100 K	10 M	100 K	
KSA27	644	13	3	1.993	94	99	33	35	1 helper virus 2 alphasatellites 1 betasatellite
KSA46	2244	19	3	3	96	97	34	34	1 helper virus
G11	1585	7	3	3	94	99	34	34	1 helper virus
Control	247	5	3	2	94	98	34	34	1 helper virus 2 betasatellites

**Table 2:** Reference-guided assembly statistics for the Illumina sequences obtained for the RCA-enriched begomovirus-satellite complexes. Reference genomes were obtained from the *de novo* assembly. CLCuGV = *Cotton leaf curl Gezira virus*, ToLCSDV = *Tomato leaf curl Sudan virus*, TYLCV-OM = *Tomato yellow leaf curl virus* from Oman, TYLCB = *Tomato* *yellow leaf curl betasatellite*, ToLCSDB = *Tomato leaf curl Sudan betasatellite*, CLCuGB = *Cotton leaf curl Gezira betasatellite*, DNA1 and DNA2 are alphasatellites.

Sample	Average Depth of Coverage		N50 (kb)		Identity of the Assembled Viral Components
	10 M	100 K	10 M	100 K	
KSA27	49,733	766	1.550	1.532	CLCuGV, CLCuGB, DNA1, DNA2
KSA46	11,853	125	2	2	ToLCSDV
G11	155,410	1480	2	2	ToLCSDV
Control	47,135	1455	1.536	1.527	TYLCV-OM, TYLCB and ToLCuSDB

**Figure 2:**
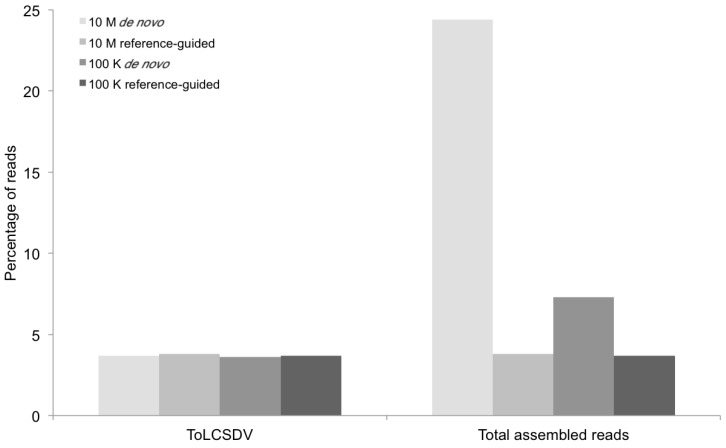
Percentage of DNA sequence reads (Y axis) mapped at different levels of sequence depth that contributed the begomoviral contigs obtained from sample KSA46 (X axis) resulting from *de novo* and reference-guided assembly. 10 M = 10 million reads, 100 K = 100 thousand reads, ToLCSDV = *Tomato leaf curl Sudan virus*.

**Figure 3:**
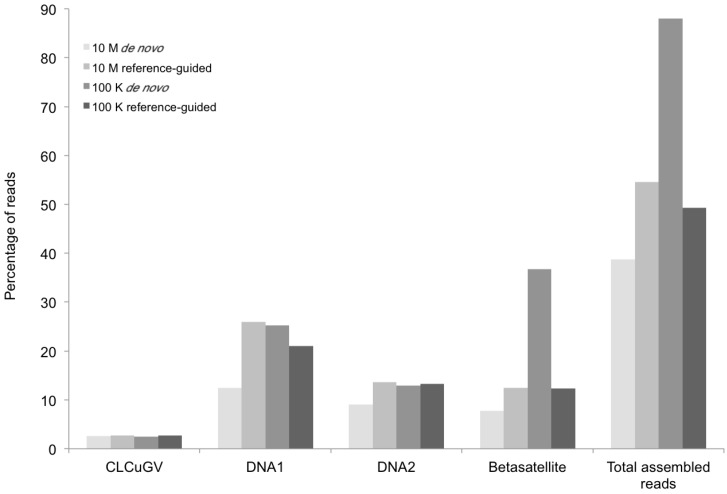
Percentage of DNA sequence reads (Y axis) mapped at different levels of sequence depth that contributed the begomoviral contigs obtained from sample KSA27 (X axis) resulting from *de novo* and reference-guided assembly. 10 M = 10 million reads, 100 K = 100 thousand reads, CLCuGV = *Cotton leaf curl Gezira virus*, DNA1 and DNA2 are new alphasatellites and betasatellites, respectively.

The BLASTn results indicated that only one kind of begomoviral genomic component (EMBL accession number HG530539) (Figure 2) was obtained from the field tomato sample (KSA46) collected in Saudi Arabia. This 2788-nt-long viral genome shared 87%–92% nt identity with several *Tomato leaf curl Sudan virus* (ToLCSDV) strains/isolates reported previously from the Nile Basin and neighboring Yemen and Oman [4,11]. The genome organization of the assembled ToLCSDV was found to be similar to other strains reported from the region [11]. However, no begomovirus-associated DNA satellite molecule was identified from this tomato field sample. In contrast, one helper virus, a betasatellite, and two types of alphasatellite (Figure 3) were found to be present in the field okra sample (KSA27), indicating that single and multiple genomic components for helper viruses and/or satellites were reliably detectable when present in begomovirus-infected field plants.

The DNA sequence comparisons indicated that the helper begomovirus, at 2780 nt in size, (EMBL Accession Number HG530540) from okra shared 92% identity with *Cotton leaf curl Gezira virus* (CLCuGV), and that the betasatellite (EMBL Accession Number HG530542) also was related to *Cotton leaf curl Gezira* *betasatellite* (CLCuGB) [7], at 61% shared nt identity. This assembled CLCuGV has genome organization similar to other CLCuGV strains reported from the Nile Basin. However, the 681-nt-long betasatellite identified in okra plant sample (KSA27) was (herein) considered to be “defective”, owing to the absence of the essential β*C*1 ORF. Because the *de novo* assembled betasatellite was divergent from its closest relatives, and it lacked the β*C*1 ORF traditional PCR was employed to amplify the target sequences and verify the identity by cloning and DNA sequencing. PCR betasatellite-specific primers [26] were used to amplify a defective molecule from total DNA extracted from the field sample, KSA46. Cloning and sequencing of the amplicons confirmed that they were identical to the *de novo* assembled molecules (EMBL Accession Number HG530542).

**Figure 4:**
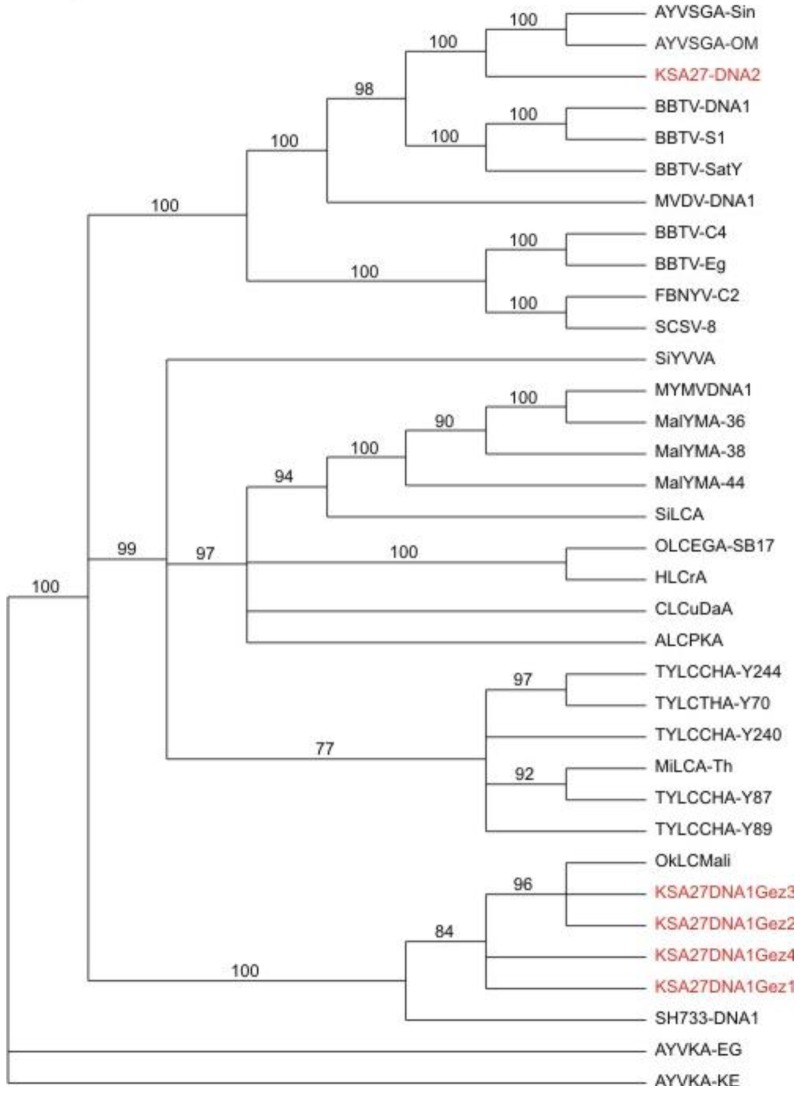
Phylogenetic relationships of alphasatellite DNA sequences obtained from field samples, and agro-inoculated positive control test plants, obtained by *de novo* assembly of the resultant DNA obtained from deep sequencing (in red), in relation to selected, reference alphasatellites. The alphasatellite acronyms and accession numbers are as described by Idris *et al*., (2011) [11].

Similarly, DNA sequence analysis of the alphasatellites obtained by *de novo* assembly from the field okra sample indicated the presence of four variants of the DNA-1 type alphasatellite at 1382 nt in size (EMBL Accession Number HG530544-HG530547), and one DNA-2 type alphasatellite at 1367 nt (EMBL Accession Number HG530543) (Figure 4) [11], are herein referred to as KSA27 DNA1 and KSA27 DNA2, respectively. These two alphasatellites contained the expected conserved ORF (Rep) on the sense strand. These KSA27 DNA1 variants shared 90%–98% identity with each other, and 30%–33% nt identity with KSA27 DNA2 isolated from the same okra plant sample. KSA27 DNA1 shared the highest nt identity with DNA1 from Mali and Gezira at 88%–94% and 86%–89%, respectively, while DNA2 shared its highest nt identity with two other DNA2 alphasatellites, reported from Oman and Singapore, at 64%. To verify the presence of the KSA27 DNA1 and DNA2 alphasatellites in plant extracts (KSA27), PCR specific primers were designed based on the *de novo* assembled alphasatellites. Specific primers for KSA27 DNA1 were: 681F-3'-TACACTCGTGGAGGATCTGC-5' and 680R-3'-GAACCAGGTCCCACTTCTGA-5', and for the KSA27 DNA2, primers were 249F-3'-GAGGAAACAACTGGCACTGG-5' and 248R-3'-CGGCGAAGGACTTAACAGAG-5'. The PCR amplicons obtained from the field-infected okra sample KSA27 using these specific primers were cloned and completely sequenced. All of the resultant clones were verified to share 100% nt identity with the genome of the *de novo*-assembled alphasatellites. The verification of satellite identities by PCR amplification, cloning, and DNA sequencing, provided robust support for the validity of the approach that involves enrichment, deep sequencing, and *de novo* assembly of the circular, ssDNA begomoviral-associated alphasatellites.

**Figure 5:**
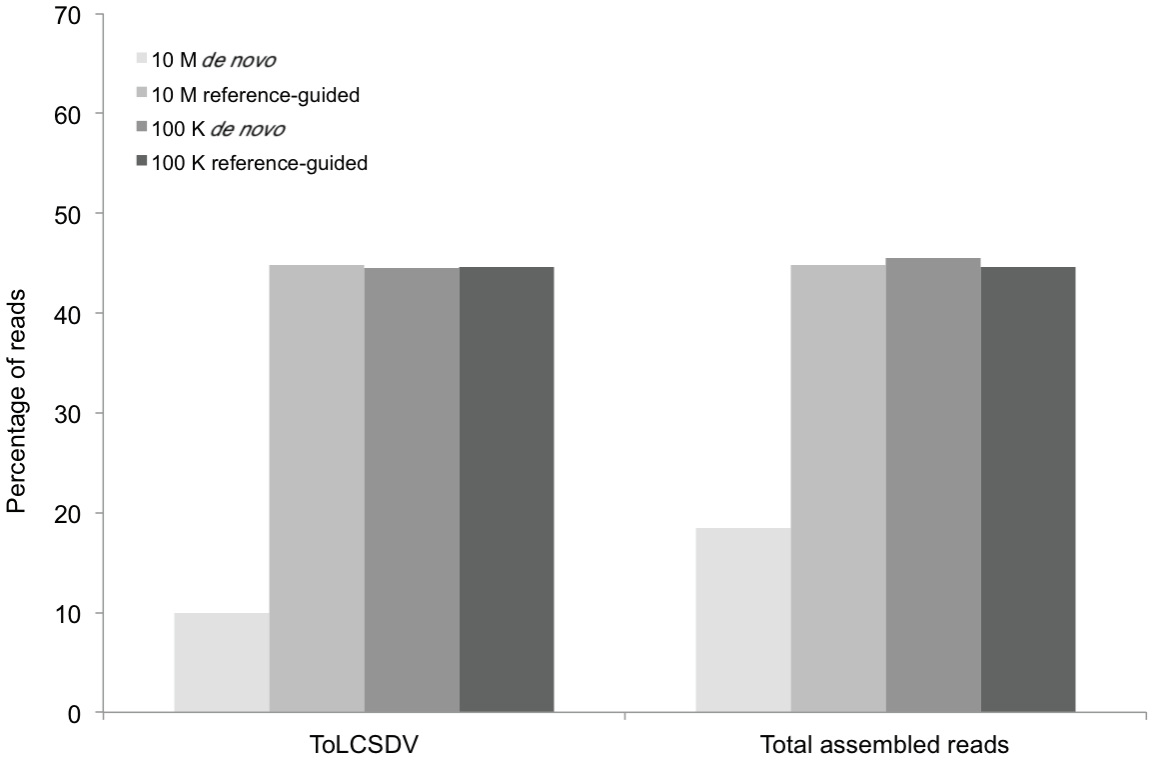
Percentage of mappable DNA sequence reads (Y axis) at different levels of sequence depth that contributed to sample G11 contigs containing the begomovirus genomic sequence (X axis) resulting from the *de novo* and reference-guided assemblies. 10 M = 10 million reads, 100 K = 100 thousand reads, ToLCSDV = *Tomato leaf curl Sudan virus*.

**Figure 6:**
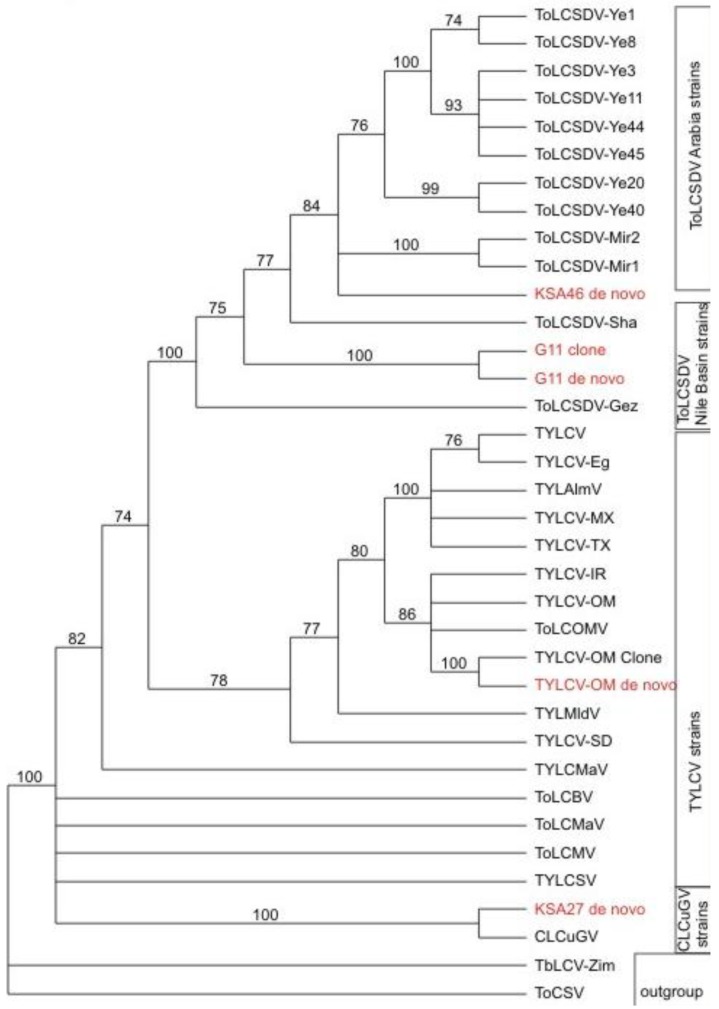
Phylogenetic relationships of helper begomovirus DNA sequences obtained from field samples, and from agro-inoculated positive control test plants, obtained by deep sequencing (in red), in relation to selected begomovirus reference sequences. The *virus* acronyms and accession numbers are as described by Idris *et al*., (2011) [11].

**Figure 7:**
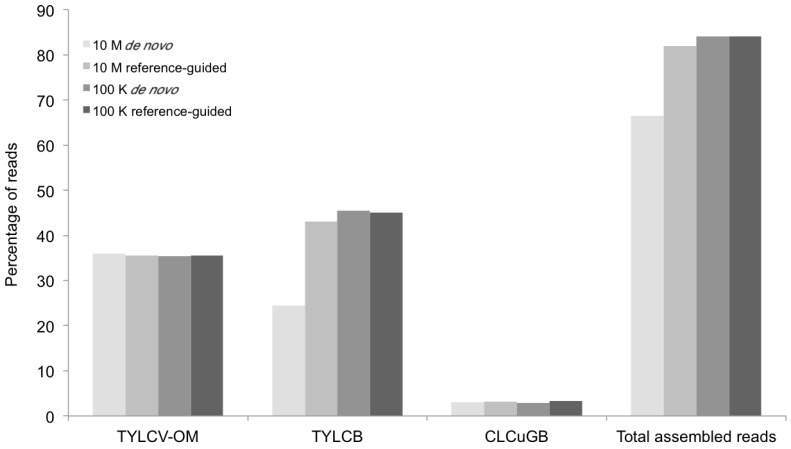
Percentage of mappable DNA sequence reads (Y axis) at different levels of sequence depth contributing to the agro-begomovirus-betatsatellite clones used to inoculate the positive control plants, and the resultant contigs containing the begomovirus genomic or betasatellite sequences (X axis) produced by the *de novo* and reference-guided assemblies. 10 M = 10 million reads, 100 K = 100 thousand reads, TYLCV-OM = *Tomato yellow leaf curl virus* from Oman, TYLCB = Tomato yellow leaf curl betasatellite, CLCuGB = Cotton leaf curl Gezira betasatellite.

A second line of support for the validity of this approach is provided by the DNA sequence analysis of two different (positive) experimental controls. The first experimental control consisted of a field-infected tomato plant sample collected from Gezira (G11) from which the single helper begomovirus, ToLCSDV, had been previously cloned (G11), and the genome sequenced using capillary DNA sequencing (GenBank Accession Number JX483705). DNA extracts from the same plant were then subjected to RCA enrichment, deep sequencing, and *de novo* assembly. A rigorous search of the contigs assembled from the deep sequencing experiment resulted in the recovery of the complete ToLCSDV genome (Figure 5 and Figure 6) (EMBL Accession Number HG530541) that was identical to the genome sequence obtained using the “traditional” approach (GenBank Accession Number JX483705). The second positive experimental control consisted of *N. benthamiana* tobacco plants inoculated (in the seedling stage) with a mixture of three *Agrobacterium* (agro)-clones of the helper virus, *Tomato yellow leaf curl virus* (TYLCV) (GenBank accession number FJ956703), the Tomato yellow leaf curl betasatellite (TYLCB) (GenBank Accession Number DQ644566), and the Cotton leaf curl Gezira betasatellite (CLCuGB) (GenBank Accession Number AY044143), from previous studies. Total DNA extracted from the agro-inoculated, symptomatic *N. benthamiana* seedlings was subjected to RCA enrichment of the begomoviral and satellite genomes present followed by deep sequencing, and *de novo* assembly. The assembled viral components and satellite molecules (Figure 6 and Figure 7) were found to share 100% nt identity with the sequences for the respective agro-clones that were used for inoculation of the tobacco plants. 

In a third line of support for the deep sequencing approach, the resultant DNA sequences that had been determined for all of the begomoviral and satellite molecules, were subjected to phylogenetic analysis. The results (Figure 6) confirmed that the helper begomoviruses associated with the KSA46 and G11 satellites, grouped together in the clade that also contained other previously determined genome sequences of ToLCSDV isolates and strains, which also clustered with a basis in known, geographical endemism (Figure 6). The helper begomovirus (associated with the KSA27 satellites DNA-1 and DNA-2) grouped into the clade containing the CLCuGV sequence. Also, the TYLCV (Figure 6) isolated from the agro-inoculated tobacco plants grouped with its closest relative, TYLCV-OM, as was expected. The two different alphasatellites obtained from sample KSA27 grouped in different clades, with the DNA-1 type clustered with other DNA1-like alphasatellites, while the second grouped with its closest DNA2 type alphasatellite relatives (Figure 4).

**Figure 8:**
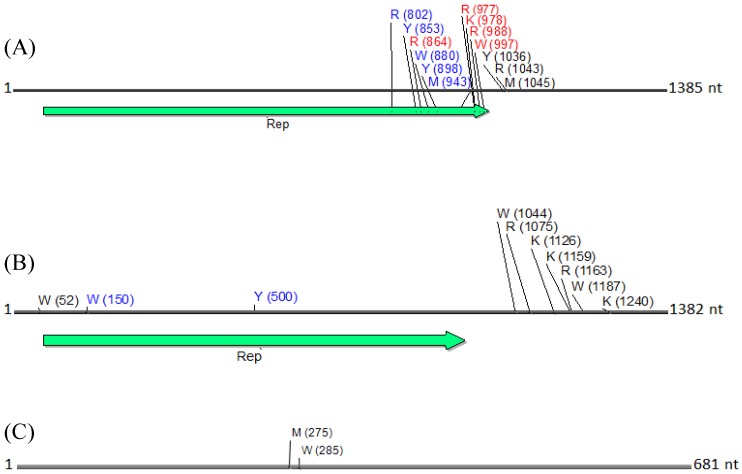
Physical maps of (**A**) DNA1, (**B**) DNA2 and (**C**) betasatellite, illustrating the locations of SNPs. SNPs are depicted as their respective nucleotide ambiguity code (Table 3), and their location on each molecule is shown in brackets. The predominant region found to contain SNPs was located in the carboxyl terminus, and immediately rightward of the C1 (Rep) ORF in a non-coding region (**A**). Non-synonymous and synonymous substitutions present in the coding region are demarcated in red and in blue, respectively, and those located in the noncoding region are shown in black. The satellite circular maps were presented as linear maps (black horizontal lines) for ease of viewing. The number of nucleotides (nt) in each molecule is indicated.

The contigs resulting from the *de novo* assemblies represented primarily concatemeric molecules consisting of repeated sequences that were also present in the respective begomovirus genome, and/or the associated satellite, making it impossible to determine the depth of coverage, and the presence and/or the precise locations of a number of SNPs (Table 3, Figure 8). To circumvent this obstacle we reassembled the short, high-throughput Illumina reads using the *de novo* assembled begomovirus genomes and satellite DNAs as reference sequences (Table 4 and Figure 2, Figure 3, Figure 5 and Figure 7). These results demonstrate a “proof of concept”, for the implementation of deep sequencing as a fast and reliable means of “harvesting” circular, ssDNAs characteristically associated with begomovirus infections in plants, and for exploring the SNPs associated with viral genomes and/or their associated satellites. Further, as few as 100,000 reads was sufficient to assemble up to four (Table 4) (and probably many more) distinct, begomoviral genomes, making the method feasible for recovering monopartite and bipartite begomoviral genomes and associated smaller circular, ssDNAs (satellites, DI’s, subgenomic molecules, others) from a single plant harboring a mixture of different viral strains or species.

The percentage of total assembled reads was variable among the begomoviruses included in this study (Figure 2, Figure 3, Figure 5 and Figure 7). This could have resulted from differences in host species, whether plants were grown in greenhouse (*N. benthamiana*) or field (okra, tomato), and perhaps also to differences between the plant species with the respect to the extent of amplification (by RCA) of mitochondria and chloroplast organelles, or to circularized chromosomal fragments. The SNPs in DNA 1 (Figure 8) were mapped to a region that spans the *C*-terminus of the satellite Rep protein (the replication associated protein), and the region located immediately downstream of this gene. Of the ten SNPs mapped in the Rep region, five resulted in an amino acid change, as a non-synonymous substitution (Figure 8). In contrast, two SNPs were present in the Rep gene of DNA2 that would result in no an amino acid change owing to a synonymous substitution (Figure 8).

**Table 3:** Identification of the position and type(s) of single nucleotide polymorphisms (SNPs) in three kinds of satellite DNA molecules assembled from next-generation sequencing reads, obtained from field sample KSA27. The reads were assembled using SeqMan NGen software that identifies SNPs and classifies them based on the International Union of Pure and Applied Chemistry code. The non-synonymous and synonymous SNPs are shown in red and in blue, respectively, in C1 ORF (see Figure 8). The SNPs in noncoding regions are shown in black. For each nucleotide position the SNPs were confirmed at ≥10 base percentage score.

DNA1			DNA2			Betasatellite		
SNP Position	SNP Type	Base percentage Score	SNP Position	SNP Type	Base Percentage Score	SNP Position	SNP Type	Base Percentage Score
802	R	A = 13; G = 87	52	W	A = 58; T = 42	275	M	A = 46; C = 54
853	Y	C = 32; T = 68	150	W	A = 56; T = 44	285	W	A = 51; T = 49
864	R	A = 64; G = 36	500	Y	C = 62; T = 38			
880	W	A = 61; T = 39	1044	W	A = 33; T = 67			
898	Y	C = 63; T = 37	1075	R	A = 77; G = 23			
943	M	A = 67; G = 33	1126	K	G = 40; T = 60			
977	R	A = 30; G = 70	1159	Y	C = 50; T = 50			
978	K	G = 30; T = 70	1163	R	A = 51; G = 49			
988	R	A = 30; G = 70	1187	W	A = 56; T = 44			
997	W	A = 69; T = 31	1240	K	G = 45; T = 55			
1036	Y	C = 69; T = 31						
1043	R	A = 32; G = 68						
1045	M	A = 33; C = 67						

**Table 4:** Genomic component size and number of SNPs. SNPs were determined based on ≤90 percent mismatches.

Sample	Genomic Components	Size (bp)	SNPs
KSA27	CLCuGV	2780	0
	CLCuGB	681	2
	DNA1	1385	13
	DNA2	1382	10
KSA46	ToLCSDV	2788	0
G11	ToLCSDV	2765	0
Control	TYLCV-OM	2767	0
	TYLCB	1371	0
	CLCuGB	1349	0

Recent advances in deep sequencing technologies, or NGS, has made it possible to utilize multiplexing to explore the composition of begomovirus populations for as many as 96 samples per flow cell lane, and to effectively reduce the cost of deep sequencing such that large numbers of virus samples can be processed simultaneously. However, for organisms with small-sized genomes [27], such as begomoviruses, the Illumina Hiseq2000/2500 is not an optimal platform because of its massive throughput capacity, unless >96 samples are multiplexed and run in a single lane [28]. In a successful 2 × 100 bp paired-end, HiSeq2000/2500 run, 30–40 Gb (150–200 million reads) of sequence are generated from one lane of a flow cell, to produce 1.56–2.08 million reads per sample, or about 112,000–149,000× coverage of each 2.8 Kb of begomovirus genome, and requires enormous time and computational capability to assemble such huge amounts of sequence data.

To minimize the sequence throughput and reduce the time devoted to computation and assembly, sequencing of begomoviral genome in an Illumina bench top sequencer, MiSeq, would likely offer a better option than the HiSeq2000/25,000 platform. In both platforms libraries need to be constructed using a high multiplexing barcode [28] in order to substantially increase the number of samples per lane to make reasonable coverage for this shot genome sizes and reduce the cost per sample. The standard MiSeq run using V3 kits can produce 25 Million reads at 3.75 Gb (2 × 75 bp PE per run) to 15 Gb (2 × 300 bp PE per run). Thus, approximately 260,000 reads can be obtained per sample with coverage of 14,000–56,000× for 96 multiplexed samples.

## 3. Experimental Section

### 3.1. Source of Begomovirus-Satellite Complexes and Viral Genomic Component Enrichment

Leaves of symptomatic tomato KSA46 (n = 1) and okra KSA27 (n = 1) plant samples were collected from Saudi Arabia during the winter, 2012. A third sample (G11) was collected from symptomatic tomato from Gezira, Sudan during the winter, 2011. The tomato plants exhibited yellow leaf curl and stunting symptoms, whereas, the okra plants showed vein thickening and leaf curl symptoms, characteristic of whitefly-transmitted begomovirus infection. Positive begomovirus infection of the field-collected tomato and okra plants was confirmed based on the results of PCR amplification, cloning, and DNA sequencing of the expected size fragment of the coat protein gene (577 base pairs) [29] (data not shown) from total DNA extracts. Preliminary identification based on DNA sequence comparisons with known begomoviruses for which sequences are available in the NCBI GenBank database indicated they were most closely related CLCuGV.

To enrich for the circular, ssDNA begomoviral genomes and associated DNA satellites, total DNA extracted from naturally infected field plants was used as a template for rolling circle amplification (RCA) [19,23], as described previously for begomoviruses [11,20,30]. Briefly, 0.5 µL of DNA template was added to 5µL of sample buffer, 5 µL of reaction mix and 0.2 µL of ɸ29 DNA polymerase and the mixture was incubated for 18 h at 30 °C, followed by incubation at 65 °C for10 min to inactivate the ɸ29 DNA polymerase. The RCA products were used as template for NGS. To assess the reliability and accuracy of NGS for begomovirus genome-satellite discovery, DNA was extracted from symptomatic *N. benthamiana* agro-inoculated with an infectious clone each of the previously characterized *Tomato yellow leaf curl virus* (TYLCV) (GenBank Accession Number FJ956703), and the DNA satellites, Tomato yellow leaf curl betasatellite (TYLCB) from Oman (GenBank Accession Number DQ644566) [11] and Cotton leaf curl Gezira betasatellite from the Nile Basin (GenBank Accession Number AY044143) [4].

### 3.2. DNA Sequencing of Genomic Components

The total DNA preparations isolated from the tomato or okra field samples (KSA27, KSA46 and G11), and from the agro-inoculated *N.* *benthamiana*, were enriched for circular DNA molecules by RCA using TempliPhi 100 kit (GE Healthcare Bio-Science, Piscataway, NJ, USA), according to the manufacturer’s instructions, and submitted for deep sequencing using the Illumina GAIIx and HiSeq2000 system (Illumina Inc., San Diego, CA, USA). The genomic shotgun libraries were prepared for Illumina sequencing using the TruSeq DNA sample preparation v. 2 kit (Illumina Inc., San Diego, CA, USA), with the standard Illumina low throughput protocol provided by the manufacturer with one minor modification, in that a PCR purification kit was used instead of the Agencourt AMPure XP (Beckman Coulter Genomics, Danvers, MA, USA) DNA clean up beads for cleaning the end-repair DNA reactions. Briefly, two micrograms of the RCA products were sheared using Adaptive Focused Acoustic Technology (Covaris Inc., Woburn, MA, USA) to produce approximately 400–500 bp fragments, which were end-repaired and purified using a Qiagen PCR clean up or Zygo Concentrator-5 kit (Zygo Research, Irvine, CA, USA). The dA nucleotides were added to the 3'-blunt end of each DNA fragment using Klenow Exo^−^ polymerase, and ligated with the dT-overhang of the multiple indexed Y-forked. The sequences of the universal primers and adapters are as indicated in the Illumina Technical note 2012.09.17 [31]. Library size separation was carried out using agarose gel electrophoresis, and the fragments were eluted, and enriched by PCR. The final size and concentration of each library was estimated using a Bioanalyzer (Agilent, Santa Clara, CA, USA) and the Qubit (Invitrogen, Carlsbad, CA, USA), respectively, and/or by qPCR analysis. Ten nM library pools were prepared by mixing the 24 libraries to achieve an equal molar concentration of each.

The pooled libraries (20 nM) were denatured in a total reaction volume of 20 uL. Seven pM of the denatured library pool was used to generate clusters in a cBot (Illumina Inc. using TruSeq PE cluster kit v3-cBot-HS, San Diego, CA, USA), according to the manufacturer’s instruction. The clustered Flow Cell was loaded into the HiSeq2000 sequencer and sequencing was carried out using a Pair-End run with 101 (Read1)-7(Index Read)-101(Read2) cycles, and the TruSeq SBS kit v3-HS (200-cycle) (Illumina Inc., San Diego, CA, USA).

### 3.3. Nucleotide Sequence Assembly

**Figure 9:**
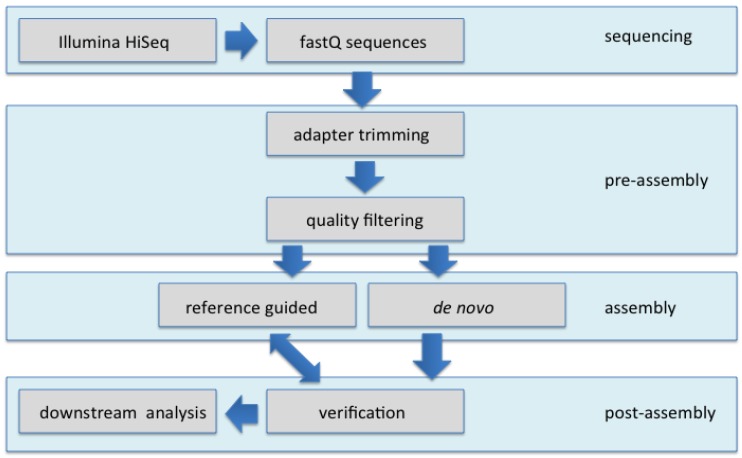
Overview of the assembly process for begomoviral genome and satellite sequences.

The Illumina short, paired end reads were subjected to standard quality trimming, de-multiplexing, and adapter removal using CASAVA 1.8.2 (Illumina Inc. San Diego, CA, USA) that also converts data into a compressed fastq format (Figure 9). The short sequences obtained were assembled using the commercially available SeqMan NGen v.3 [32]. This software uses distance information from the paired-read sequences (library sizes) to link short contigs into larger scaffolds. All contigs >600 nt long obtained from SeqMan NGen analyses were annotated using the BLASTn algorithm available at the NCBI GenBank website to search the non-redundant (nr) GenBank database using the Net Search option build-in the software. A BLASTn search was carried out for 10 contigs at a time to determine the predicted identity of the assembled fragments. To further verify the identity of the assembled satellite DNAs, satellite-specific PCR primers were designed and used for amplification followed by cloning and Sanger sequencing. To locate viral SNPs, the full-length viral genomes obtained from the *de novo* assembly were used as references for “reference-guided” assembly (Table 2 and Table 4; Figure 1). Each nucleotide position marked as a SNP when it had a quality score that was greater than the threshold value, and if the mismatch was at least 10% at each position (Table 3). The begomoviral genome and satellite molecules assembled from the NGS reads were used to reconstruct a phylogenetic tree for each respective type of molecule obtained, using maximum parsimony (MP) with the parameters, as previously described [11].

## 4. Conclusions

Metagenomics has been used successfully to determine the genome sequence and associated SNPs of selected begomoviral genomes, and their associated satellites from total DNA extracts of symptomatic tomato and okra plants, without cloning or any previous knowledge of the target virus-satellite complexes that were present in naturally-infected plants from the field. By first enriching for the circular ssDNA begomoviral and satellite molecules using RCA, and subjecting those products to Illumina deep sequencing followed by *de novo* assembly, the begomoviral genomes and satellite DNA molecules identified from these plant samples were the same as those found to be present using conventional methods. Two types of experimental controls were implemented to validate the RCA-NGS approach. These involved Illumina sequencing of libraries constructed from a tomato field sample (G11) and laboratory-grown *N. benthamiana* plants experimentally-inoculated with a mixture of the helper begomovirus and betasatellites for which clones were previously constructed and the DNA sequence determined.

The naturally infected tomato plant sample (G11) from which one begomovirus-ToLCSDV was isolated, cloned, and characterized following RCA, molecular cloning, and capillary (Sanger) sequencing. Total DNA from this same sample was also subjected to RCA enrichment of the suspected begomovirus(es) and their associated satellites, followed by deep sequencing and *de novo* assembly. A single helper virus genome sequence of one virus was obtained from both approaches.

The second experimental control was prepared by agro-inoculating laboratory grown *N. benthamiana* plants with a mixture of previously cloned begomoviral-satellite molecules [11] of TYLCV-OM, TYLCB, and CLCuGB. The total DNA extracts isolated from the symptomatic *N. benthamiana* plants were processed using the new approach including RCA enrichment, Illumina sequencing, and *de novo* assembly, which, based on the assembled sequences, confirmed that all three genomic components that were used for agro-inoculation of *N. benthamiana* plants were present. The subsequently determined sequence of helper viral genome TYLCV-OM and the associated betasatellites, TYLCB, and CLCuGB, were aligned and compared to their respective sequence counterparts previously determined for the begomoviral-satellite clones, and were found to be the identical.
